# Public Health Response to *Aedes aegypti* and *Ae. albopictus* Mosquitoes Invading California, USA

**DOI:** 10.3201/eid2110.150494

**Published:** 2015-10

**Authors:** Charsey Cole Porse, Vicki Kramer, Melissa Hardstone Yoshimizu, Marco Metzger, Renjie Hu, Kerry Padgett, Duc J. Vugia

**Affiliations:** California Department of Public Health, Sacramento, Richmond, and Ontario, California, USA

**Keywords:** Invasive Aedes mosquitoes, Aedes albopictus, Aedes aegypti, chikungunya fever, dengue, dengue virus, chikungunya virus, California, mosquitoes, vectors, viruses, public health response, surveillance, education, control

## Abstract

*Aedes aegypti* and *Ae. albopictus* mosquitoes, primary vectors of dengue and chikungunya viruses, were recently detected in California, USA. The threat of potential local transmission of these viruses increases as more infected travelers arrive from affected areas. Public health response has included enhanced human and mosquito surveillance, education, and intensive mosquito control.

Two mosquito species, *Aedes aegypti* and *Ae. albopictus,* are principal vectors for dengue virus (DENV) and chikungunya virus (CHIKV), both infectious to humans. These mosquitoes are daytime biters, need only small water containers to propagate, and are difficult to eradicate. 

Dengue, caused by DENV infection, is a viral febrile illness characterized by headache, retro-orbital pain, myalgia, arthralgia, rash, and potentially hemorrhagic manifestations. Most persons with dengue are asymptomatic, but the virus can still be transmitted to mosquitoes that bite them (*1*). Reported dengue-associated illnesses and deaths are high, and increasing worldwide; an estimated 50–100 million cases occur annually (*2*). Since 2000, DENV transmission has occurred in areas of the United States where *Ae. aegypti* and *Ae. albopictus* are established, and outbreaks have occurred in Florida, Texas, and Hawaii (*3*).

Chikungunya fever, caused by CHIKV infection, is also a viral febrile illness and is characterized by severe polyarthralgia (*4*,*5*). Most persons infected with CHIKV are symptomatic (*4*,*6*). An ongoing outbreak of CHIKV began in late 2013 in the Caribbean and has spread to 43 countries or territories throughout Central America, South America, North America, and the Caribbean (*7*). As of 2014, more than 2,000 cases of chikungunya fever had been reported in the United States; except for 11 locally acquired cases in Florida, all cases were acquired abroad (*8*).

DENV- and CHIKV-infected returned travelers or visitors presumably imported the viruses and served as sources for local US outbreaks (*3*,*6*). The established presence of *Ae. aegypti* and *Ae. albopictus* mosquitoes in the southeastern United States poses a threat for potential transmission of DENV and CHIKV to local residents as infected, possibly viremic, travelers return or arrive from countries where transmission is ongoing. This threat is now expanding to California. In several regions of the state, imported cases of DENV and CHIKV infections have been documented where recent detection and persistence of *Ae. albopictus* and *Ae. aegypti* mosquitoes concurrently occur.

## The Study

In California, surveillance and control of mosquitoes and mosquitoborne diseases is a collaborative effort involving vector-control agencies, local health departments, the California Department of Public Health (CDPH), and the University of California, Davis, Center for Vectorborne Diseases (CVEC). Vector-control agencies perform mosquito surveillance and control by using specialized traps and products targeting adult and immature mosquitoes. Local health departments conduct follow-up of reported human cases. CDPH provides confirmation of mosquitoborne disease cases in humans, mosquito identification, and consultation and assistance in responding to invasive mosquitoes and disease trends. CVEC tests *Ae. aegypti* and *Ae. albopictus* mosquitoes for DENV, CHIKV, and West Nile virus.

Before 2011, *Ae. aegypti* and *Ae. albopictus* mosquitoes were rarely detected in California and were not known to persist. Of these previous detections, the largest was an infestation of *Ae. albopictus* mosquitoes that were imported with “lucky bamboo” from China into Los Angeles County in 2001 (*9*). In 2011, *Ae. albopictus* mosquitoes were detected in El Monte, Los Angeles County, California, and genetic analysis indicated they may have been remnants of the 2001 importation (*10*). Since 2011, enhanced mosquito surveillance has documented the persistence and spread of *Ae. albopictus* mosquitoes to 12 surrounding cities within Los Angeles County.

In 2013, *Ae. aegypti* mosquitoes were detected in Fresno, Madera, and San Mateo Counties, California; analysis indicated they were genetically most similar to *Ae. aegypti* mosquitoes from the southeastern United States (*11*). In 2014, *Ae. aegypti* mosquitoes persisted in those 3 counties and were also detected in Kern, Tulare, Los Angeles, and San Diego Counties. In 2015, *Ae. aegypti* mosquitoes were detected in Imperial, Orange, and Alameda Counties (Figure). Of 1,729 *Ae. aegypti* mosquitoes captured during 2013–2014 and tested at CVEC, none were positive for DENV or CHIKV.

**Figure:**
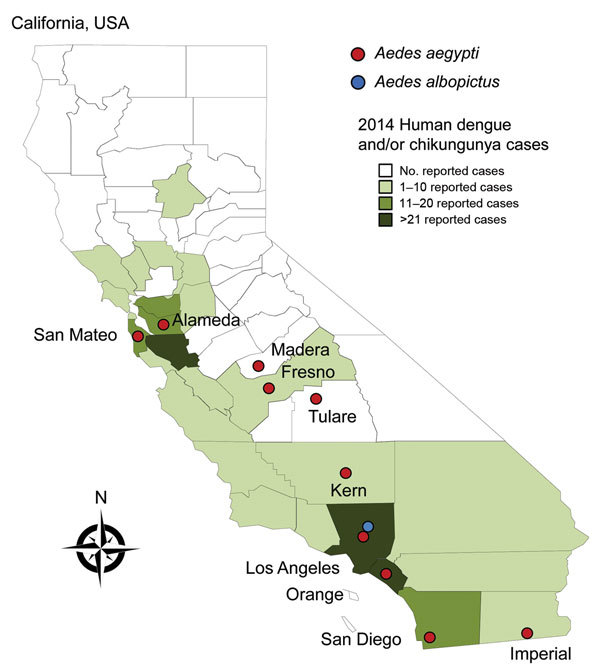
Invasive *Aedes* mosquitoes detected during 2011–2015 and number of imported human cases of dengue, chikungunya fever, or both reported during 2014 in counties in California, USA.

Dengue, but not chikungunya fever, is reportable in California. Nonetheless, CDPH is notified of confirmed and probable chikungunya fever cases. During 2009–2013, CDPH was notified of 3 confirmed chikungunya fever cases, and the reported annual number of dengue cases increased from 36 to 125, all among returned travelers or visitors. In 2014, a total of 141 chikungunya fever and 133 dengue cases were reported, all in persons with recent travel to DENV- or CHIKV-endemic or outbreak areas. Of the 133 dengue case-patients, 98 (74%) were likely viremic while in California, 71 (54%) had illness onset after arrival, and 27 (20%) had illness onset within 5 days before arrival. Of the 141 chikungunya fever case-patients, 93 (66%) were likely viremic in California, 58 (41%) had illness onset after arrival, and 35 (25%) had illness onset within 7 days before arrival. Of these likely viremic patients, 44% (43/98) with dengue and 58% (54/93) with chikungunya fever arrived or returned to a county with an infestation of invasive *Aedes* mosquitoes and, thus, represented a potential risk for virus transmission if bitten by an *Ae. aegypti* or *Ae. albopictus* mosquito.

## Discussion

We document the invasion and persistence in California of *Ae. albopictus* mosquitoes since 2011 and *Ae. aegy*pti mosquitoes since 2013. The risk for local transmission of DENV and CHIKV is currently low because of small numbers of infected travelers arriving in California and limited distribution of invasive *Aedes* mosquitoes in the state. However, the threat for local transmission of these viruses is increasing as more infected, potentially viremic travelers arrive from affected areas. The presence of *Ae. albopictus* and *Ae. aegypti* mosquitoes in southern California is of particular concern because of the state’s large population, high number of travelers, and proximity to Mexico. Of the DENV and CHIKV infections reported in 2014, a total of 59% (162/274) were in southern California residents. In 2014, Mexico reported 32,100 dengue and 155 chikungunya fever cases (*12*,*13*), and the presence of *Ae. aegypti* mosquitoes has been established in Mexican cities along the California–Mexico border (e.g., Mexicali, Tecate, Tijuana) (*14*). The large number of persons crossing the US–Mexico border each day creates another potential source of imported cases of dengue and chikungunya fever.

In response to the threat of increased transmission of these viruses, CDPH has developed and distributed guidance to local public health and vector-control agencies to enhance human case and mosquito surveillance, increase knowledge and awareness of these diseases among the general public and the local medical community, and apply intensive *Aedes* mosquito control and prevention measures (*15*). Continuous enhanced surveillance for human cases of DENV and CHIKV infection and ongoing monitoring of invasive *Aedes* mosquito populations are necessary to gauge the level of risk in various regions of the state. Surveillance and monitoring is also necessary to increase the public health response if an *Aedes* mosquito tests positive for DENV or CHIKV or if a patient without travel consistent for DENV or CHIKV exposure has laboratory-confirmed dengue or chikungunya fever. Improved public awareness will increase reporting of mosquitoes that bite during the day, and improved awareness among health care providers may shorten the time to diagnosis of dengue and chikungunya fever cases. Targeted mosquito surveillance and control are essential for limiting the density and geographic spread of these mosquitos to minimize the risk of a viremic person coming in contact with them. Close collaboration between CDPH and local health departments and vector-control agencies is necessary to obtain accurate and timely travel histories from case-patients and to implement follow-up mosquito surveillance in areas surrounding their residences. Through these public health activities, we hope to keep the risk for local transmission of DENV and CHIKV low while the threat is increasing.
